# Bolsa Família program and incomplete childhood vaccination in two Brazilian cohorts

**DOI:** 10.11606/s1518-8787.2020054001774

**Published:** 2020-11-04

**Authors:** Francelena de Sousa Silva, Rejane Christine de Sousa Queiroz, Maria dos Remédios Freitas Carvalho Branco, Vanda Maria Ferreira Simões, Yonna Costa Barbosa, Marcelo Augusto Ferraz Ruas do Amaral Rodrigues, Marco Antonio Barbieri, Heloísa Bettiol, Maria da Conceição Pereira Saraiva, Luiz Guilherme Scorzafave, Maria Isabel Accoroni Theodoro Habenschus, Antônio Augusto Moura da Silva

**Affiliations:** I Secretaria Municipal de Saúde São LuísMA Brasil Secretaria Municipal de Saúde. São Luís, Maranhão, MA, Brasil; II Universidade Federal do Maranhão Departamento de Saúde Pública São LuísMA Brasil Universidade Federal do Maranhão. Departamento de Saúde Pública. Programa de Pós-Graduação em Saúde Coletiva. São Luís, Maranhão, MA, Brasil; III Universidade Federal do Maranhão Departamento de Patologia São LuísMA Brasil Universidade Federal do Maranhão. Departamento de Patologia. Programa de Pós-Graduação em Saúde Coletiva. São Luís, Maranhão, MA, Brasil; IV Universidade Federal do Maranhão Hospital Universitário Presidente Dutra São LuísMA Brasil Universidade Federal do Maranhão. Hospital Universitário Presidente Dutra. São Luís, Maranhão, MA, Brasil; V Hospital Sarah São LuísMA Brasil Hospital Sarah. São Luís, Maranhão, MA, Brasil; VI Universidade de São Paulo Faculdade de Medicina de Ribeirão Preto Ribeirão PretoSão Paulo Brasil Universidade de São Paulo. Faculdade de Medicina de Ribeirão Preto. Programa de Pós-Graduação em Saúde da Criança e do Adolescente. Ribeirão Preto, São Paulo, Brasil; VII Universidade de São Paulo Faculdade de Economia Departamento de Economia Ribeirão PretoSão Paulo Brasil Universidade de São Paulo. Faculdade de Economia. Departamento de Economia. Programa de Pós-Graduação em Economia Aplicada. Ribeirão Preto, São Paulo, Brasil

**Keywords:** Vaccination Coverage, Poverty, Social Programs, Child health

## Abstract

**OBJECTIVE:**

To estimate the effect of being a beneficiary of the *Bolsa Família* Program (BFP) in the vaccination of children aged 13 to 35 months.

**METHODS:**

Our study was based on all birth records of residents of Ribeirão Preto (SP) and probabilistic sampling with 1/3 of the births of residents of São Luís (MA), selecting low-income children, born in 2010, belonging to the cohorts Brazilian Ribeirão Preto and São Luís Birth Cohort Studies and eligible for the *Bolsa Família* program. The information of *Cadastro Único* (CadÚnico – Single Registry) was used to categorize the receipt of benefit from the BFP (yes or no). The final sample consisted of 532 children in Ribeirão Preto and 1,229 in São Luís. The outcome variable was a childhood vaccine regimen, constructed with BCG, tetravalent, triple viral, hepatitis B, poliomyelitis, rotavirus and yellow fever vaccines. The adjustment variables were: economic class, mother's schooling and mother's skin color. Children with monthly per capita family income of up to R$ 280.00 and/or economic class D/E were considered eligible for the benefit of the BFP. A theoretical model was constructed using a directed acyclic graph to estimate the effect of being a beneficiary of the BFP in the vaccination of low-income children. In the statistical analyses, weighing was used by the inverse of the probability of exposure and pairing by propensity score.

**RESULTS:**

Considering a monthly per capita family income of up to R$ 280.00, being a beneficiary of the BFP had no effect on the childhood vaccination schedule, according to weighing by the inverse of the probability of exposure (SL-coefficient: −0.01; 95%CI −0.07 to 0.04; p = 0.725 and RP-coefficient: 0.04; 95%CI −0.02 to 0.10; p = 0.244) and pairing by propensity score (SL-coefficient: −0.01; 95%CI −0.07 to 0.05; p = 0.744 and RP-coefficient: 0.04; 95%CI −0.02 to 0.10; p = 0.231).

**CONCLUSIONS:**

The receipt of the benefit of the BFP did not influence childhood vaccination, which is one of the conditionalities of the program. This may indicate that this conditionality is not being adequately monitored.

## INTRODUCTION

Childhood vaccination positively impacts children's health by favoring the eradication, elimination, prevention and control of several immunopreventable diseases that still cause significant infant morbidity and mortality worldwide^1^. Policies that reduce inequalities in the vaccination situation are essential. In Brazil, the *Bolsa Família* Program (BFP), a public policy for conditional income transfer to Brazilians in poverty and extreme poverty, stands out^2^.

BFP adopts as eligibility criteria for receiving the benefit the monthly per capita family income and family composition, being eligible families with pregnant, nursing mothers, children and/or adolescents. The families contemplated must comply with some conditionalities: school attendance for children and adolescents, prenatal care for pregnant women, monitoring of child growth and development and compliance with the National Calendar of Vaccination of Children^2^.

Some studies that assess the relationship between receiving the benefit of BFP and childhood vaccination, especially comparing regions with different socioeconomic conditions^3^^,^^4^. We could not find comparing the data surveyed with information from the *Cadastro Único* (*CadÚnico* – Single Registry) of the Ministry of Social Development for social programs of the Brazilian Federal Government.

Shei, et al.^3^ found a positive association between receiving BFP benefit and greater vaccination coverage in low-income children. However, a study by Andrade, et al.^4^ did not verify such association^4^.

Considering the importance of childhood vaccination and that this is one of the conditionalities to be a beneficiary of the BFP, and also considering the scarcity of studies, the divergence of results and the lack of studies comparing the data with information from *CadÚnico*, our study aimed to analyze the effect of being a beneficiary of the BFP in the vaccination of children.

## METHODS

### Study design

This study used data from the Brazilian Ribeirão Preto and São Luís Birth Cohort Studies (BRISA), developed in two moments: birth (2010) and first follow-up (2011 to 2013), from 13 to 35 months of age. All children had already completed one year of age, having the opportunity to receive all vaccines planned for that age. Data from both municipalities were used in both moments.

### Study Population and Sample

**Figure 1:**
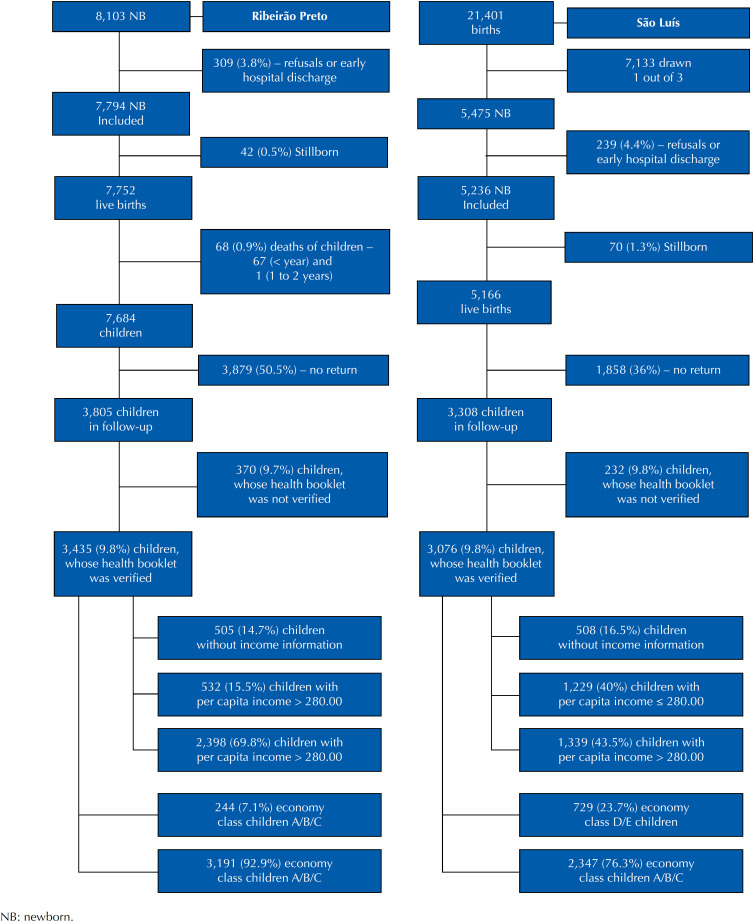
Sample flowcharts of children with per capita income of up to R$ 280.00/economic class D/E belonging to the BRISA birth cohort, at birth and follow-up in children under 3 years of age, Ribeirão Preto (SP) and São Luís (MA), Brazil, 2010–2013.

In Ribeirão Preto, the BRISA birth cohort included all deliveries of women living in the city that occurred at least in the prior three months in hospital units in 2010. For our study, we selected only children that met the eligibility criteria to receive the *Bolsa Família* benefit. The final sample consisted of 532 children from families with monthly per capita income of up to R$ 280.00, a proxy for the eligibility criterion. Due to the known income information problems, we also used as a *proxy* for the eligibility criterion “belonging to class D or E,” according to the economic classification of the *Associação Brasileira de Estudos e Pesquisas* (ABEP – Brazilian Association of Research Enterprises)^5^. Thus, by this second criterion, we selected 244 children belonging to families of economic class D or E, aged 13 to 35 months (Figure 1).

In São Luís, the BRISA birth cohort was composed of a probabilistic sample of births in hospital units in 2010, with more than 100 deliveries/year, representing 94.7% of these deliveries. The births of newborns (NB) from families living in the municipality for at least three months were randomly selected with a sample interval of one in three births. The sampling was systematic and stratified proportionally to the number of deliveries per hospital^6^. For our study, we selected only children that met the eligibility criterion to receive the BF benefit. Therefore, the final samples were of 1,229 children from families with monthly per capita income of up to R$ 280.00 and 729 children belonging to families of economic class D or E, aged 13 to 35 months (Figure 1).

### Variables and Theoretical Model

**Figure 2:**
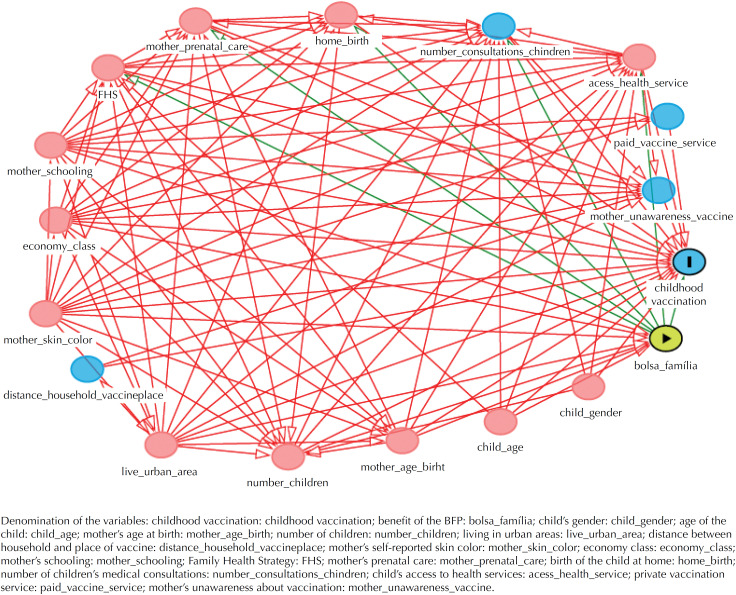
Directed acyclic graph showing the effect of the benefit of the *Bolsa Família* Program (BFP) on childhood vaccination.

The theoretical model used to analyze the effect of being a beneficiary of the BFP in childhood vaccination^3^^,^^4^^,^^7^^–^^17^ was constructed using a directed acyclic graph (Figure 2), using the DAGitty software (version 2.0 alpha, Johannes Textor). Based on the graph, the assumptions of the relationships between the variables were assumed and the implications of testable independences were derived. The variables identified to compose the minimum set of sufficient adjustment for confounding, based on the criterion of the back door^18^, were: economic class, mother's skin color (self-reported) and mother's schooling.

### Outcome Variable

The outcome variable, collected at the time of follow-up, was a “childhood vaccination schedule,” categorized as complete and incomplete. For its construction, the seven vaccines that must be taken in the first year of life were used as parameters, according to the National Calendar of Vaccination of children of the Brazilian Ministry of Health in force since the beginning of 2010. Therefore, we considered one dose for BCG vaccine, three for hepatitis B vaccine, two for rotavirus vaccine, three for polio vaccine, three for tetravalent vaccine, one for yellow fever vaccine and one for viral triple vaccine^19^. Each child had their vaccination schedule categorized as complete or incomplete, according to the recommendations of the *Programa Nacional de Imunização* (PNI – National Immunization Program). If the child no longer received the recommended number of doses for at least one of the seven vaccines, his vaccination schedule was considered incomplete.

### Exposure Variable

The exposure variable, also collected at the time of follow-up, was called “BFP beneficiary” and categorized as “yes” or “no”. In addition to the self-reported data of the cohorts, we also identified: information about the receipt of the BFP, from the *CadÚnico* database, and benefit value, in the BFP database, both referring to the period from 2011 to 2013. The data were obtained in accordance with the process regulated by Article 11 of Ordinance 10/2012 of the Ministry of Social Development^20^.

The databases were compared by probabilistic pairing with STATA software (version 14.0, StataCorp). In the *CadÚnico* database, we found 2,057 of the 3,308 children from São Luís, and 1,033 of the 3,805 children from Ribeirão Preto. The variables name and date of birth of the child's mother were used as link keys between the databases and the cohorts, *CadÚnico* and payment of the benefits of BFP. The information from the *CadÚnico* database was used to define if the child was a beneficiary or not of BFP and the payment information to identify the values of the benefits.

Information on income, when used in isolation, may present inconsistencies^21^, such as ignorance or omission of income by the informant, which may result in underestimated income values^22^. To reduce this limitation, two variables were used as a proxy for the eligibility criterion for the child to be a beneficiary of BFP, similarly to the study by Schmidt, et al.^23^.

The first variable was monthly per capita family income, obtained through the variables number of residents in the household and declared monthly family income. In 2010, the eligibility criterion of the child to receive the BF benefit was to belong to the family of monthly per capita income of up to R$ 140.00. However, for our study, a monthly per capita family income of up to R$ 280.00 was considered as a proxy for the eligibility criterion, to encompass a greater number of low-income children benefiting from BFP and increase the accuracy of the estimates.

Moreover, the database included children with monthly per capita family income between R$ 140.00 and R$ 280.00 that received the benefit of the BFP. Thus, we considered that this value (up to R$ 280.00) still includes low-income children, corroborating studies that also defined cut-off points of monthly per capita family income higher than the eligibility criterion of the child to receive the benefit of BFP^3^^,^^4^.

The BFP benefit value was included in the cohort information on monthly family income. The value of the benefit – obtained in research in the database – was subtracted from family income.

The other variable used as a proxy for the eligibility criterion was economic class, categorized in A/B, C and D/E, according to strata of the Brazil Criterion, of ABEP, in force from 2010 to 2013^5^. Some economic level indicators tend to be more stable, showing smaller changes over time and less probability of measurement error for household classification. One of these indicators is the economic class, which includes household goods and the head of education^21^. Children that belonged to the poorest families with lower purchasing power, belonging to classes D and E, were considered eligible to receive the benefit of BFP.

### Adjustment variables

- Economy class: A/B, C or D/E. Used in the adjustment only when the eligibility criterion for receiving the BF was monthly per capita family income.
- Mother's schooling (years of study): ≥ 12, 9 to 11 and 0 to 8.
- Mother's skin color (self-reported): white, brown or black.

The adjustment variables were obtained from the cohorts at birth.

### Statistical Analysis

Absolute and relative frequencies were estimated for the adjustment, exposure and outcome variables. To estimate the effect of being a beneficiary of the BFP in childhood vaccination and to verify the consistency of the results, two estimation procedures were used: pairing by propensity score by the nearest neighbor method and weighting by the inverse of the probability of exposure. First, the predictive model of exposure (BFP beneficiary) was estimated in a multiple logistic regression model, verifying the probability of each participant being a beneficiary of the BFP (this probability is called “propensity score”). This model included the variables economic class and mother's skin color and schooling.

Subsequently, the multiple linear regression explanatory model was estimated to analyze the effect of exposure on the outcome using the teffects ipwra (inverse-probability-weighted regression adjustment) and teffects psmatch (propensity-score matching) routines in the Stata program. In the explanatory model, the coefficients and their respective confidence intervals are interpreted as a difference in the percentage of incomplete vaccination between the groups of beneficiaries and non-beneficiaries of BFP. In this model, only the variable “to be a beneficiary of the BFP” was included as an explanatory variable.

The chi-square test was used to estimate the percentages of participation in the follow-up according to various characteristics. The estimates were also weighted by the inverse of the probability of selection due to differences in the percentage of follow-up according to some variables. In the logistic model, the probabilities of participation in the follow-up were estimated as a function of the predictor variables. The final weight of the weighting process was the multiplication of the inverse of the probability of having participated in the follow-up according to the predictor variables of participation by the inverse of the probability of receiving benefit from BFP, depending on the predictor variables of receiving the benefit (the propensity score).

To verify the balance between the groups (non-beneficiary children and BFP beneficiaries that belonged to families with monthly per capita income of up to R$ 280.00 and/or class D/E) compared to the adjustment variables, we performed tests through the tebalance summ routine, obtaining the estimates: standardized absolute differences between the means (between −0.2 and 0.2) and variance ratio (between 0.9 and 1.1)^24^.

We verified if there was a common support area through the distribution of the propensity score in beneficiaries and non-beneficiaries of the BFP in boxplot. We adopted a 5% significance level and 95% confidence intervals (95%CI). For the analyses, we used Stata statistical package (version 14.0).

### Ethical aspects

This research was approved by the Research Ethics Committee of the Hospital Universitário Clementino Fraga Filho, Universidade Federal do Rio de Janeiro, opinion No. 223/2009-30. This study was approved by the Research Ethics Committee of *Hospital das Clínicas of Faculdade de Medicina de Ribeirão Preto* of *Universidade de São Paulo*, protocol no. 4.116/2008.

## RESULTS

The percentage of children belonging to low-income families (up to R$ 280.00) that did not receive the benefit of BFP was higher in Ribeirão Preto (41.3%) than in São Luís (29.1%) (Table 1). Of these children, in São Luís, 3.6% had a monthly per capita family income of up to R$ 70.00, 22.6% from R$ 71.00 to R$ 140.00, and 73.8% from R$ 141.00 to R$ 280.00. In Ribeirão Preto, these percentages were 2.3% up to R$ 70.00, 13.7% from R$ 71.00 to R$ 140.00 and 84% from R$ 141.00 to R$ 280.00 (data not shown in table).

**Table 1:** Percentages of vaccination incompleteness, receipt of the *Bolsa Família* Program benefit and adjustment variables of low-income children, from 13 to 35 months of age, in the birth cohorts BRISA, Ribeirão Preto (SP) and São Luís (MA), Brazil, 2010–2013.

Variables			São Luís				Ribeirão Preto			
			N (3.076)^e^	%	N (1.229)^f^	%	N (3.435)^e^	%	N (532)^f^	%
Vaccine incompleteness^a^										
	BCG Vaccine		17	0.6	9	0.7	72	2.1	10	1.9
	Polio vaccine		135	4.4	74	6.0	98	2.8	15	2.8
	Hepatitis B vaccine		178	5.8	63	5.9	101	2.9	14	2.6
	Tetravalent vaccine		251	8.2	103	8.4	155	4.5	17	3.2
	Yellow fever vaccine		310	10.1	129	10.5	128	3.7	16	3.0
	Triple viral vaccine		341	11.1	153	12.5	155	4.5	28	5.3
	Human rotavirus vaccine		591	19.2	287	23.3	227	6.6	54	10.1
	Childhood vaccination schedule^b^		1,045	33.9	460	37.4	422	12.3	81	15.2
Exposure variable										
	Beneficiary of the *Bolsa Família* Program									
		No	1,432	46.5	358	29.1	2,683	78.2	219	41.3
		Yes	1,644	53.5	871	70.9	749	21.8	311	58.7
Adjustment variables										
	Economy class^c^									
		A/B	565	18.4	42	3.4	1,597	46.5	69	12.9
		C	1,782	57.9	713	58.0	1,594	46.4	353	66.4
		D/E	729	23.7	474	38.6	244	7.1	110	20.7
	Mother's schooling in years									
		> 12	419	13.8	27	2.3	745	22.0	9	1.7
		9–11	2,244	73.8	951	78.1	2,172	64.1	334	63.9
		0–8	380	12.4	239	19.6	470	13.9	179	34.3
	Mother's skin color^d^									
		White	539	17.7	151	12.4	2,005	59.2	219	41.9
		Brown	2,089	68.8	888	72.3	1,045	30.9	218	41.7
		Black	409	13.5	182	14.9	336	9.9	86	16.4

Variables			São Luís (n = 1,229)^f^				Ribeirão Preto (n = 532)^f^			
			n (358)^g^	%	n (871)^h^	%	n (219)^g^	%	n (311)^h^	%
Vaccine incompleteness^a^										
	BCG Vaccine		02	0.6	7	0.8	06	2.7	04	1.3
	Polio vaccine		27	7.5	47	5.4	09	4.1	06	1.9
	Hepatitis B vaccine		21	5.9	42	4.8	08	3.6	06	1.9
	Tetravalent vaccine		34	9.5	69	7.9	08	3.6	09	2.9
	Yellow fever vaccine		42	11.7	87	9.9	08	3.6	08	2.6
	Triple viral vaccine		46	12.8	107	12.3	09	4.1	19	6.1
	Human rotavirus vaccine		88	24.6	199	22.8	18	8.2	36	11.6
	Childhood vaccination schedule^b^		134	37.4	326	37.4	27	12.3	54	17.4
Adjustment variables										
	Economy class^c^									
		A/B	20	5.6	22	2.6	41	18.7	28	9.0
		C	208	58.1	505	57.9	139	63.5	213	68.5
		D/E	130	36.3	344	39.5	39	17.8	70	22.5
	Mother's schooling in years									
		> 12	13	3.7	14	1.6	05	2.3	04	1.3
		9–11	277	78.2	674	78.1	158	73.5	175	57.4
		0–8	64	18.1	175	20.3	52	24.2	126	41.3
	Mother's skin color^d^									
		White	50	14.0	101	11.7	110	50.9	108	35.3
		Brown	258	72.3	630	72.9	73	33.8	145	47.4
		Black	49	13.7	133	15.4	33	15.3	53	17.3

Differences between the sums of absolute values and sample, due to lost information; BRISA: Brazilian Ribeirão Preto and São Luís Birth Cohort Studies.

a Incomplete vaccination according to parameters of the Ministry of Health (MH).

b Incomplete childhood vaccination schedule: not having received at least one dose of BCG vaccine, three for hepatitis B, three for poliomyelitis, three for tetravalent, one for yellow fever, one for triple viral and two for human rotavirus. Vaccines from the first year of life, which were part of the National Calendar of Vaccination of Children in early 2010.

c economic classification according to the *Associação Brasileira de Estudos e Pesquisas* (ABEP – Brazilian Association of Research Enterprises)

d Mother's skin color (self-reported).

e Total number of children at the time of follow-up in children under 3 years, with a health booklet verified.

f Children belonging to families with monthly per capita income of up to R$ 280.00.

g Children belonging to families with monthly per capita income of up to R$ 280.00 non-beneficiaries of the BFP.

h Children belonging to families with monthly per capita income of up to R$ 280.00 beneficiaries of the BFP.

In São Luís, the percentage of incompleteness of the childhood vaccination scheme in low-income children was the same among beneficiaries (37.4%) and non-beneficiaries (37.4%), whereas in Ribeirão Preto the percentage was higher among beneficiaries (17.4%) when compared with non-beneficiaries (12.3%) (Table 1).

Both in São Luís and Ribeirão Preto, among children belonging to low-income families (per capita family income of up to R$ 280.00), being a beneficiary of the BFP had no effect on the childhood vaccination schedule, according to weighting by the inverse of the probability of exposure (São Luís – coefficient: −0.01; 95%CI −0.07 – 0.04; p = 0.708; and Ribeirão Preto – coefficient: 0.04; 95%CI −0.02 – 0.10; p = 0.218) and pairing by propensity score (São Luís – coefficient: −0.01; 95%CI −0.07 – 0.05; p = 0.744; and Ribeirão Preto – coefficient: 0.04; 95%CI −0.02 – 0.10; p = 0.231).

Among children belonging to the families of classes D/E, in both municipalities, being a beneficiary of the BFP also had no effect on the childhood vaccination schedule, according to weighting by the inverse of the probability of exposure (São Luís – coefficient: −0.04; 95CI% −0.11 – 0.03; p = 0.288; and Ribeirão Preto – coefficient: −0.01; 95%CI −0.11 – 0.08; p = 0.827) and pairing by propensity score (São Luís – coefficient: −0.04; 95%CI −0.11 – 0.03; p = 0.312; and Ribeirão Preto – coefficient: −0.01; 95%CI −0.11 – 0.09; p = 0.820).

The BFP also had no effect on childhood vaccination when each vaccine was analyzed individually (BCG vaccine, hepatitis B, human rotavirus, poliomyelitis, tetravalent, triple viral and yellow fever) (Table 2).

**Table 2:** Estimates for the effect of being a beneficiary of the *Bolsa Família* Program in the vaccination of low-income children (monthly per capita family income of up to R$ 280.00/economic class D/E), from 13 to 35 months of age. Birth cohorts BRISA, Ribeirão Preto (SP) and São Luís (MA), Brazil, 2010–2013.

Vaccine incompleteness	Children belonging to families with monthly per capita income of up to R$ 280.00. Beneficiary of the Bolsa Família Program							
	São Luís (N = 1.229)				Ribeirão Preto (N = 532)			
	Weighing by the inverse of the probability of exposure		Propensity score pairing		Weighing by the inverse of the probability of exposure		Propensity score pairing	
	Coefficient (IC95%)	p	Coefficient (IC95%)	p	Coefficient (IC95%)	p	Coefficient (IC95%)	
Childhood vaccination schedule^a^	-0.01 (-0.07 – 0.04)	0.708	-0.01 (-0.07 – 0.05)	0.744	0.04 (-0.02 – 0.10)	0.218	0.04 (-0.02 – 0.10)	0.231
BCG Vaccine^b^	-0.01 (-0.01 – 0.00)	0.634	-0.01 (-0.01 – 0.00)	0.795	0.01 (-0.01 – 0.04)	0.265	0.01 (-0.01 – 0.04)	0.264
Hepatitis B vaccine^c^	0.01 (-0.01 – 0.04)	0.405	0.01 (-0.00 – 0.04)	0.370	0.01 (-0.01 – 0.04)	0.291	0.01 (-0.01 – 0.04)	0.368
Human rotavirus vaccine^d^	0.02 (-0.02 – 0.08)	0.289	0.03 (-0.02 – 0.08)	0.260	-0.02 (-0.07 – 0.02)	0.365	-0.02 (-0.07 – 0.02)	0.357
Polio vaccine^e^	0.02 (-0.00 – 0.05)	0.101	0.02 (-0.00 – 0.05)	0.097	0.01 (-0.01 – 0.04)	0.303	0.01 (-0.01 – 0.04)	0.305
Tetravalent vaccine^f^	0.02 (-0.01 – 0.05)	0.260	0.02 (-0.01 – 0.06)	0.196	-0.01 (-0.03 – 0.03)	0.963	-0.01 (-0.03 – 0.03)	0.975
Triple viral vaccine^g^	0.01 (-0.03 – 0.05)	0.595	0.01 (-0.03 – 0.05)	0.594	-0.01 (-0.05 – 0.02)	0.425	-0.01 (-0.05 – 0.03)	0.566
Yellow fever vaccine^h^	0.02 (-0.01 – 0.06)	0.210	0.02 (-0.01 – 0.06)	0.181	0.01 (-0.02 – 0.04)	0.516	0.01 (-0.02 – 0.04)	0.517

Vaccine incompleteness	Children belonging to families of classes D/E Beneficiary of the *Bolsa Família* Program							
	São Luís (N = 729)				Ribeirão Preto (N = 244)			
	Weighing by the inverse of the probability of exposure		Propensity score pairing		Weighing by the inverse of the probability of exposure		Propensity score pairing	
	Coeficiente (IC95%)	p	Coeficiente (IC95%)	p	Coeficiente (IC95%)	p	Coeficiente (IC95%)	p
Childhood vaccination schedule^a^	-0.04 (-0.11 – 0.03)	0.288	-0.04 (-0.11 – 0.03)	0.312	-0.01 (-0.11 – 0.08)	0.827	-0.01 (-0.11 – 0.09)	0.820

BCG Vaccine^b^	0.01 (-0.00 – 0.01)	0.556	0.01 (-0.00 – 0.01)	0.528	0.02 (-0.00 – 0.05)	0.080	0.02 (-0.00 – 0.05)	0.082
Hepatitis B vaccine^c^	0.01 (-0.02 – 0.04)	0.551	0.01 (-0.02 – 0.04)	0.571	0.03 (-0.01 0.07)	0.197	0.02 (-0.01 – 0.07)	0.231
Human rotavirus vaccine^d^	0.03 (-0.03 – 0.11)	0.263	0.03 (-0.03 – 0.11)	0.258	0.02 (-0.06 – 0.10)	0.611	0.02 (-0.06 – 0.11)	0.572
Polio vaccine^e^	0.01 (-0.02 – 0.05)	0.568	0.01 (-0.02 – 0.05)	0.606	0.03 (-0.00 – 0.07)	0.053	0.03 (-0.00 – 0.07)	0.055
Tetravalent vaccine^f^	0.03 (-0.01 – 0.08)	0.160	0.03 (-0.01 – 0.07)	0.187	0.01 (-0.04 – 0.07)	0.555	0.01 (-0.04 – 0.07)	0.631
Triple viral vaccine^g^	0.03 (-0.01 – 0.08)	0.213	0.03 (-0.01 – 0.09)	0.187	0.06 (-0.00 – 0.11)	0.062	0.06 (-0.00 – −0.11)	0.064
Yellow fever vaccine^h^	0.05 (-0.00 – 0.11)	0.061	0.05 (-0.00 – 0.11)	0.058	0.02 (-0.01 0.05)	0.225	0.02 (-0.01 – 0.05)	0.238

BRISA: Brazilian Ribeirão Preto and São Luís Birth Cohort Studies; 95%CI confidence interval with a 5% significance level.

a Incomplete childhood vaccination schedule: not having received at least one dose of BCG vaccine, three for hepatitis B, three for poliomyelitis, three for tetravalent, one for yellow fever, one for triple viral and two for human rotavirus. Vaccines from the first year of life, which were part of the National Calendar of Vaccination of Children in early 2010.

b Incomplete BCG vaccine: not having received at least one dose.

c Incomplete hepatitis B vaccine: not having received at least three doses.

d Incomplete human rotavirus vaccine: not having received at least two doses.

e Incomplete polio vaccine: not having received at least three doses.

f Incomplete tetravalent vaccine: not having received at least three doses.

g Incomplete triple viral vaccine: not having received at least one dose.

h Incomplete yellow fever vaccine: not having received at least one dose.

In both municipalities, when analyzing children, whose families had a monthly per capita family income of up to R$ 140.00 as a criterion for eligibility for the benefit of the BFP (according to the Ministry of Social Development), or the total number of children in the sample, being a beneficiary of BFP also had no effect on the childhood vaccination schedule and for each vaccine alone.

For the childhood vaccination schedule, the balance between the groups of beneficiaries and non-beneficiaries of the BFP was achieved by the two eligibility criteria used for all adjustment variables, suggesting an interchangeability between the groups regarding the variables observed (Table 3). Balance was also obtained between the groups in the other analyses. The Boxplot has shown the existence of a common support area between beneficiaries and non-beneficiaries of BFP.

**Table 3:** Standardized differences and variance ratios of the adjustment variables to estimate the effect of being a beneficiary of the *Bolsa Família* Program in the vaccination of children belonging to families with monthly per capita income of up to R$ 280.00/economic class D/E, from 13 to 35 months of age, in the birth cohorts BRISA, São Luís (MA) and Ribeirão Preto (SP), Brazil, 2010–2013.

Adjustment variables		Children belonging to families with monthly per capita income of up to R$ 280.00 beneficiaries of the BFP.					
		Gross		Weighing by the inverse of the probability of survival		Propensity score pairing	
		Standardized difference	Variance ratio	Standardized difference	Variance ratio	Standardized difference	Variance ratio
São Luís (n = 1.229)							
Economy class^a^							
	A/B						
	C	-0.01	1.00	-0.00	1.00	-0.00	1.00
	D/E	0.07	1.03	0.00	1.00	0.00	1.00
Mother's schooling in years							
	> 12						
	9–12	-0.00	1.00	-0.00	1.00	-0.00	1.00
	0–8	0.05	1.09	0.00	1.00	0.00	1.00
Mother's skin color^b^							
	White						
	Brown	0.01	0.98	0.00	0.99	0.00	0.99
	Black	0.04	1.08	-0.00	0.99	0.00	1.00
Ribeirão Preto (n = 532)							
Economy class^a^							
	A/B						
	C	0.11	0.92	-0.00	1.00	-0.01	1.00
	D/E	0.09	1.15	0.00	1.00	0.00	1.00
Mother's schooling in years							
	> 12						
	9–12	-0.34	1.24	0.01	0.99	-0.00	1.00
	0–8	0.36	1.31	-0.01	0.99	0.01	1.00
Mother's skin color^b^							
	White						
	Brown	0.28	1.11	-0.01	0.99	-0.00	0.99
	Black	0.05	1.10	0.01	1.02	0.00	1.00

Adjustment variables		Children belonging to families with monthly per capita income of up to R$ 280.00 beneficiaries of the BFP.					
		Gross		Weighing by the inverse of the probability of survival		Propensity score pairing	
		Standardized difference	Variance ratio	Standardized difference	Variance ratio	Standardized difference	Variance ratio
São Luís (n = 729)							
Mother's schooling in years							
	> 12						
	9–12	0.01	0.98	0.00	0.99	0.00	1.00
	0–8	0.05	1.06	-0.00	0.99	0.00	1.00
Mother's skin color^2^							
	White						
	Brown	-0.02	1.02	-0.00	0.99	-0.00	1.00
	Black	0.08	1.18	0.00	0.99	0.00	1.00
Ribeirão Preto (n = 244)							
Mother's schooling in years							
	> 12						
	9–12	-0.28	1.23	-0.00	1.00	-0.00	1.00
	0–8	0.30	1.28	0.00	1.00	0.00	1.00
Mother's skin color^2^							
	White						
	Brown	0.24	0.99	0.00	1.00	0.02	1.00
	Black	0.14	1.32	0.00	1.00	0.02	1.00

BRISA: Brazilian Ribeirão Preto and São Luís Birth Cohort Studies.

a Economic classification according to the *Associação Brasileira de Estudos e Pesquisas* (ABEP – Brazilian Association of Research Enterprises)

b Mother's skin color (self-reported).

## DISCUSSION

In our study, we observed that being a beneficiary of the *Bolsa Família* program had not affected childhood vaccination in children belonging to low-income families, both in São Luís and Ribeirão Preto.

One of the limitations of this research is the selection bias due to losses in the follow-up of the cohort. However, we sought to reduce this possible bias by weighting the estimates also by the inverse of the probability of participation in the follow-up, in addition to the propensity score. Another possible limitation is the confounding bias by omitted variable. Despite the use of the directed acyclic graph to represent the theoretical model, if this model does not reflect reality, the adjustment performed using the variables identified by the back door criterion may not have been sufficient to remove the confusion. However, we consider the possibility of confounding by an omitted or inadequately specified variable (e.g., occupation of the head of household) to be small, because we included in the adjustment three variables for measuring socioeconomic status, which is the main confounding of the association studied.

Among the strengths of the study, we point out the comparative analysis between two municipalities with different socioeconomic conditions, which gives more consistency to the results.

Pairing was used based on the propensity score and weighting by the inverse of the probability of exposure to evaluate the effect of BFP on vaccination of low-income children^3^^,^^4^ and reduce confounding bias. Different from the weighting by the inverse of the probability of exposure, the pairing by propensity score tends to present greater internal validity and lower external validity of the data^18^.

Despite the lower percentage of low-income children not contemplated by the BFP in São Luís (29.1%) compared to Ribeirão Preto (41.3%), the percentage of incompleteness of the childhood vaccination schedule, also in low-income children, was higher in São Luís (37.4%) compared with Ribeirão Preto (15.2%). The percentage of incompleteness, however, was high in both municipalities. In general, comparatively poorer regions, such as São Luís, have more low-income families contemplated by BFP^25^ and higher vaccination incompleteness^8^^–^^11^^,^^13^^,^^14^^,^^16^^,^^26^.

Vaccination incompleteness is higher in low-income children, and receiving the benefit of the BFP did not influence childhood vaccination, either for each vaccine alone or for all of them, in both municipalities. Compliance with the National Child Vaccination Calendar is one of the conditionalities for children to keep being a beneficiary of BFP^2^. However, this monitoring may not be effective^25^. BFP does not seem to be able to improve childhood vaccination, which is an important health indicator^4^. Either conditionality is not being adequately observed, or perhaps only it alone is not sufficient to ensure vaccination coverage if other actions are not implemented, such as the expansion of primary care and the availability of vaccines in health centers.

Another study with low-income children with representativeness for three large areas of Brazil (Northeast, Southeast/South and North/Midwest regions), found no influence of BFP on childhood vaccination^4^. In our study, propensity score was used in the statistical analysis. However, the research was conducted in the second year of implementation of *Bolsa Família* (2005), when the program had not yet undergone moments of great expansion, and monitoring of health conditionalities was still being implemented. Our study was conducted from 2011 to 2013, when the program was already consolidated.

Other studies found results different from those of our investigation, with a positive association between receiving income-conditioned transfer program benefit and greater infant vaccination coverage^3^^,^^27^. The study by Shei, et al.^3^ also evaluated the BFP and used a propensity score in its statistical analyses; however, unlike our study, it had no municipal coverage, as it was restricted to a low-income community in Salvador. The authors also emphasized that the research participants were linked to a local health center, which may have favored access to health services and, consequently, a better monitoring of conditionalities, including childhood vaccination.

A demographic and health survey conducted in India from 2007 to 2008, with children aged 12 to 23 months, used a propensity score and identified an increase in childhood vaccination rates in children benefiting from a conditional income transfer program. In the Indian study, reported vaccination information was also considered, in addition to data from children that presented proof of vaccination status. The effect of the conditional income transfer program on vaccination tended to disappear when only data of children with immunization cards were considered^27^, suggesting that the positive association observed occurred due to measurement bias. In our study, only vaccination data recorded on the child's card were considered.

A conditional income transfer program that has demonstrated a possible increase in the use of preventive health services, including childhood vaccination^28^, is the Opportunities program in Mexico, which has improved health outcomes, growth and child development. The performance of the program results from a more effective control of conditionalities, including those related to health, by a structured information system that accompany beneficiary families. The benefit is transferred to the families each two months, but only occur if the conditionalities are met by the beneficiaries^29^.

We showed that being a beneficiary of the BFP did not influence the vaccination percentages of low-income children in two Brazilian municipalities located in two regions with different socioeconomic conditions. Therefore, it is important to improve both the monitoring of the conditionality of the program and the monitoring of the vaccination situation, since the percentages of vaccine incompleteness in children benefiting from the BFP were high.
