# Supplementary material for: Bolsa Família program and incomplete childhood vaccination in two Brazilian cohorts
Source: Rev Saude Publica. 2020 Nov 4;54:98. doi: 10.11606/s1518-8787.2020054001774 (PMC7575218; doi:10.11606/s1518-8787.2020054001774)
Supplement: Translation

# Translation: Programa bolsa família e vacinação infantil incompleta em duas coortes brasileiras

**Authors:** Francelena de Sousa Silva, Rejane Christine de Sousa Queiroz, Maria dos Remédios Freitas Carvalho Branco, Vanda Maria Ferreira Simões, Yonna Costa Barbosa, Marcelo Augusto Ferraz Ruas do Amaral Rodrigues, Marco Antonio Barbieri, Heloísa Bettiol, Maria da Conceição Pereira Saraiva, Luiz Guilherme Scorzafave, Maria Isabel Accoroni Theodoro Habenschus, Antônio Augusto Moura da Silva

**Affiliations:** I São LuísMA Brasil Secretaria Municipal de Saúde. São Luís, Maranhão, MA, Brasil; II São LuísMA Brasil Universidade Federal do Maranhão. Departamento de Saúde Pública. Programa de Pós-Graduação em Saúde Coletiva. São Luís, Maranhão, MA, Brasil; III São LuísMA Brasil Universidade Federal do Maranhão. Departamento de Patologia. Programa de Pós-Graduação em Saúde Coletiva. São Luís, Maranhão, MA, Brasil; IV São LuísMA Brasil Universidade Federal do Maranhão. Hospital Universitário Presidente Dutra. São Luís, Maranhão, MA, Brasil; V São LuísMA Brasil Hospital Sarah. São Luís, Maranhão, MA, Brasil; VI Ribeirão PretoSão Paulo Brasil Universidade de São Paulo. Faculdade de Medicina de Ribeirão Preto. Programa de Pós-Graduação em Saúde da Criança e do Adolescente. Ribeirão Preto, São Paulo, Brasil; VII Ribeirão PretoSão Paulo Brasil Universidade de São Paulo. Faculdade de Economia. Departamento de Economia. Programa de Pós-Graduação em Economia Aplicada. Ribeirão Preto, São Paulo, Brasil

**Keywords:** Cobertura Vacinal, Pobreza, Programas Sociais, Saúde da Criança

## Abstract

**OBJETIVO:**

Estimar o efeito de ser beneficiário do Programa Bolsa Família (PBF) na vacinação de crianças de 13 a 35 meses.

**MÉTODOS:**

Partiu-se de todos os registros de nascimentos de residentes de Ribeirão Preto (SP) e de amostragem probabilística com ⅓ dos nascimentos de residentes de São Luís (MA), selecionando-se crianças de baixa renda, nascidas em 2010, pertencentes às coortes *Brazilian Ribeirão Preto and São Luís Birth Cohort Studies* e elegíveis ao PBF. As informações do Cadastro Único (CadÚnico) foram utilizadas para categorizar o recebimento de benefício do PBF (sim ou não). A amostra final foi de 532 crianças em Ribeirão Preto e 1.229 em São Luís. A variável-desfecho foi esquema vacinal infantil, construída com as vacinas BCG, tetravalente, tríplice viral, hepatite B, poliomielite, rotavírus e febre amarela. As variáveis de ajuste foram: classe econômica, escolaridade da mãe e cor de pele da mãe. Consideraram-se elegíveis ao benefício do PBF crianças com renda familiar *per capita* mensal de até R$ 280,00 e/ou da classe econômica D/E. Para estimar o efeito de ser beneficiário do PBF na vacinação de crianças de baixa renda, construiu-se um modelo teórico por meio de gráfico acíclico direcionado. Nas análises estatísticas, foi usada ponderação pelo inverso da probabilidade de exposição e pareamento por escore de propensão.

**RESULTADOS:**

Considerando renda familiar *per capita* mensal de até R$ 280,00, ser beneficiário do PBF não teve efeito no esquema vacinal infantil, segundo ponderação pelo inverso da probabilidade de exposição (SL-coeficiente: −0,01; IC95% −0,07 a 0,04; p = 0,725 e RP-coeficiente: 0,04; IC95% −0,02 a 0,10; p = 0,244) e pareamento pelo escore de propensão (SL-coeficiente: −0,01; IC95% −0,07 a 0,05; p = 0,744 e RP-coeficiente: 0,04; IC95% −0,02 a 0,10; p = 0,231).

**CONCLUSÕES:**

O recebimento do benefício do PBF não exerceu influência sobre a vacinação infantil, que é uma das condicionalidades do programa. Isso pode indicar que essa condicionalidade não está sendo adequadamente acompanhada.

## INTRODUÇÃO

A vacinação infantil impacta positivamente a saúde das crianças ao favorecer a erradicação, eliminação, prevenção e controle de diversas doenças imunopreveníveis, que ainda acarretam significativa morbimortalidade infantil no mundo^1^. Políticas que minimizem as desigualdades na situação vacinal são fundamentais. No Brasil, destaca-se o Programa Bolsa Família (PBF), política pública de transferência condicionada de renda aos brasileiros em situação de pobreza e extrema pobreza^2^.

O PBF adota como critérios de elegibilidade para o recebimento do benefício a renda familiar *per capita* mensal e a composição familiar, sendo elegíveis as famílias com gestante, nutriz, criança e/ou adolescente. As famílias contempladas ficam obrigadas a cumprir algumas condicionalidades: frequência escolar para crianças e adolescentes, realização de prénatal para as gestantes, acompanhamento do crescimento e desenvolvimento infantil e cumprimento do Calendário Nacional de Vacinação da Criança^2^.

São poucos os trabalhos que avaliam a relação entre o recebimento do benefício do PBF e a vacinação infantil, especialmente comparando regiões com diferentes condições socioeconômicas^3^^,^^4^. Não foram encontrados estudos que comparassem os dados coletados por meio de pesquisa com as informações do Cadastro Único (CadÚnico) do Ministério do Desenvolvimento Social para programas sociais do Governo Federal.

Shei et al^3^. encontraram associação positiva entre receber benefício do PBF e maior cobertura vacinal infantil em crianças de baixa renda. Entretanto, o estudo de Andrade et al.^4^ não verificou tal associação^4^.

Considerando a importância da vacinação infantil e que esta é uma das condicionalidades para ser beneficiário do PBF, e considerando ainda a escassez de estudos, a divergência de resultados e a ausência de pesquisa que compare os dados com informações do CadÚnico, o presente estudo objetivou analisar o efeito de ser beneficiário do PBF na vacinação de crianças.

## MÉTODOS

### Delineamento do Estudo

Este estudo usou dados das coortes de nascimentos *Brazilian Ribeirão Preto and São Luís Birth Cohort Studies* (BRISA), desenvolvidas em dois momentos: nascimento (2010) e primeiro seguimento (2011 a 2013), dos 13 aos 35 meses de idade. Todas as crianças já tinham completado um ano de idade, tendo a oportunidade de receber todas as vacinas previstas para essa idade. Foram utilizados os dados dos dois municípios, nos dois momentos.

### População e Amostra do Estudo

Em Ribeirão Preto, a coorte de nascimento BRISA incluiu todos os partos de mulheres residentes no município ocorridos há no mínimo três meses, em unidades hospitalares, no ano de 2010. Para este estudo, foram selecionadas apenas crianças que preenchiam o critério de elegibilidade para recebimento do benefício do PBF. Com isso, a amostra final foi de 532 crianças de famílias com renda *per capita* mensal de até R$ 280,00, *proxy* do critério de elegibilidade. Devido aos conhecidos problemas de informação da renda, foi usado também como *proxy* do critério de elegibilidade pertencer à classe D ou E, segundo a classificação econômica da Associação Brasileira de Empresas de Pesquisa (Abep)^5^. Assim, por este segundo critério, foram selecionadas 244 crianças pertencentes a famílias de classe econômica D ou E, na faixa etária de 13 a 35 meses de idade (Figura 1).

**Figura 1:** Fluxogramas amostrais de crianças com renda *per capita* de até R$ 280,00/classe econômica D/E pertencentes à coorte de nascimento BRISA, ao nascimento e no seguimento em menores de 3 anos, Ribeirão Preto (SP) e São Luís (MA), Brasil, 2010–2013.

Em São Luís, a coorte de nascimento BRISA foi composta por amostra probabilística dos nascimentos ocorridos em unidades hospitalares em 2010, com mais de 100 partos/ano, representando 94,7% desses partos. Os nascimentos de recém-nascidos (RN) de famílias residentes no município há no mínimo três meses foram sorteados com intervalo amostral de um a cada três nascimentos. A amostragem foi sistemática e estratificada proporcionalmente ao número de partos por hospital^6^. Neste estudo, foram selecionadas apenas crianças que preenchiam *proxy* do critério de elegibilidade para recebimento do benefício do PBF. Com isso, as amostras finais foram de 1.229 crianças de famílias com renda *per capita* mensal de até R$ 280,00 e 729 crianças pertencentes a famílias de classe econômica D ou E, na faixa etária de 13 a 35 meses de idade (Figura 1).

### Variáveis e Modelo Teórico

O modelo teórico utilizado para analisar o efeito de ser beneficiário do PBF na vacinação infantil^3^^,^^4^^,^^7^^–^^17^ foi construído por meio de gráfico acíclico direcionado (Figura 2), utilizando-se o *software* DAGitty (versão 2.0 alfa, Johannes Textor). A partir do gráfico foram assumidos os pressupostos das relações entre as variáveis e derivadas as implicações de independências testáveis. As variáveis identificadas para compor o conjunto mínimo suficiente de ajuste para confundimento, com base no critério da porta de trás^18^, foram: classe econômica, cor de pele da mãe (autorrelatada) e escolaridade da mãe.

**Figura 2:** Gráfico acíclico direcionado referente ao efeito do benefício do Programa Bolsa Família (PBF) na vacinação infantil.

### Variável-Desfecho

A variável-desfecho, coletada no momento do seguimento, foi “esquema vacinal infantil”, categorizado em completo e incompleto. Para sua construção, foram utilizadas as sete vacinas que devem ser tomadas no primeiro ano de vida, de acordo com o Calendário Nacional de Vacinação da Criança do Ministério da Saúde vigente desde o início do ano de 2010. Portanto, considerou-se como esquema completo: uma dose para a vacina BCG, três para vacina contra a hepatite B, duas para vacina contra o rotavírus, três para vacina contra a poliomielite, três para tetravalente, uma para vacina contra a febre amarela e uma para tríplice viral^19^. Cada criança teve seu esquema vacinal categorizado como completo ou incompleto, de acordo com o recomendado pelo Programa Nacional de Imunização (PNI). Se a criança deixou de receber o número de doses preconizadas para pelo menos uma das sete vacinas, seu esquema vacinal foi considerado incompleto.

### Variável de Exposição

A variável de exposição, também coletada no momento do seguimento, foi denominada “beneficiário do PBF” e categorizada em sim ou não. Além dos dados autorreferidos das coortes, também foram identificados: informações sobre recebimento do PBF, por meio do banco de dados do CadÚnico, e valor do benefício, no banco de dados do PBF, ambos referentes ao período de 2011 a 2013. Os dados foram obtidos em conformidade com o processo regulado pelo artigo 11 da Portaria 10/2012 do Ministério do Desenvolvimento Social^20^.

Os bancos de dados foram comparados por meio de pareamento probabilístico, com o *software* STATA (versão 14.0, StataCorp). No banco de dados do CadÚnico, foram encontradas 2.057 das 3.308 crianças de São Luís, e 1.033 das 3.805 crianças de Ribeirão Preto. Foram utilizadas as variáveis nome e data de nascimento da mãe da criança como chaves de ligação entre os bancos de dados e as coortes, CadÚnico e de pagamento dos benefícios do PBF. Utilizaram-se as informações do banco de dados do CadÚnico para definir se a criança era beneficiária ou não do PBF e as de pagamento para identificar os valores dos benefícios.

As informações sobre renda, quando utilizadas de forma isolada, podem apresentar inconsistências^21^, como desconhecimento ou omissão de rendimentos pelo informante, o que pode resultar em valores de rendimento subestimados^22^. Para reduzir esta limitação, empregaram-se duas variáveis como *proxy* do critério de elegibilidade para a criança ser beneficiária do PBF, de modo semelhante ao estudo de Schmidt et al^23^.

A primeira variável foi renda familiar *per capita* mensal, obtida por meio das variáveis número de residentes no domicílio e renda familiar mensal declarada. Em 2010, o critério de elegibilidade da criança para recebimento do benefício do PBF era pertencer a família de renda *per capita* mensal de até R$ 140,00. Contudo, para o presente estudo, foi considerada renda familiar *per capita* mensal de até R$ 280,00 como *proxy* do critério de elegibilidade, a fim de contemplar um maior número de crianças de baixa renda beneficiárias do PBF e aumentar a precisão das estimativas.

Além disso, no banco de dados, constavam crianças com renda familiar *per capita* mensal entre R$ 140,00 e R$ 280,00 que recebiam o benefício do PBF. Desta forma, considerou-se que esse valor (até R$ 280,00) ainda contempla crianças de baixa renda, corroborando estudos que também definiram pontos de corte de renda familiar *per capita* mensal maiores que o critério de elegibilidade da criança para recebimento do benefício do PBF^3^^,^^4^.

O valor do benefício do PBF estava incluso na informação das coortes sobre renda familiar mensal. O valor do benefício – obtido em pesquisa no banco de dados – foi subtraído da renda familiar.

A outra variável utilizada como *proxy* do critério de elegibilidade foi classe econômica, categorizada em A/B, C e D/E, segundo estratos do Critério Brasil, da ABEP, em vigor nos anos de 2010 a 2013^5^. Alguns indicadores de nível econômico tendem a ser mais estáveis, apresentando menores mudanças ao longo do tempo e menor probabilidade de erro de aferição para a classificação das famílias. Um destes indicadores é a classe econômica, que contempla bens domésticos e grau de escolaridade do chefe de família^21^. Foram consideradas como elegíveis ao recebimento do benefício do PBF crianças que pertenciam às famílias mais pobres, com menor poder de compra, pertencentes às classes D e E.

### Variáveis de Ajuste

- Classe econômica: A/B, C ou D/E. Utilizada no ajuste apenas quando o critério de elegibilidade ao recebimento do PBF foi renda familiar *per capita* mensal.
- Escolaridade da mãe (anos de estudo): ≥ 12, 9 a 11, 0 a 8.
- Cor de pele da mãe (autorreferida): branca, parda ou preta.

As variáveis de ajuste foram obtidas das coortes, ao nascimento.

### Análise Estatística

Estimaram-se frequências absolutas e relativas para as variáveis de ajuste, exposição e desfecho. Para estimar o efeito de ser beneficiário do PBF na vacinação infantil e verificar a consistência dos resultados, foram utilizados dois procedimentos de estimação: pareamento por escore de propensão pelo método do vizinho mais próximo e ponderação pelo inverso da probabilidade de exposição. Primeiramente, estimou-se o modelo preditivo da exposição (beneficiário do PBF) em modelo de regressão logística múltipla, verificando-se a probabilidade de cada participante ser beneficiário do PBF (essa probabilidade é denominada “escore de propensão”). Nesse modelo foram incluídas as variáveis classe econômica, cor da pele e escolaridade materna.

Posteriormente, estimou-se o modelo explicativo de regressão linear múltipla para análise do efeito da exposição no desfecho por meio das rotinas *teffects ipwra* (*inverse-probability-weighted regression adjustment*) e *teffects psmatch (propensity-score matching*), no programa Stata. No modelo explicativo, os coeficientes e seus respectivos intervalos de confiança são interpretados como diferença no percentual de vacinação incompleta entre os grupos de beneficiários e não beneficiários do PBF. Nesse modelo, apenas a variável “ser beneficiário do PSF” foi incluída como explicativa.

O teste do qui-quadrado foi utilizado para estimar os percentuais de participação no seguimento de acordo com várias características. Como houve diferenças no percentual de seguimento de acordo com algumas variáveis, as estimativas também foram ponderadas pelo inverso da probabilidade de seleção. No modelo logístico, foram estimadas as probabilidades de participação no seguimento em função das variáveis preditoras. O peso final do processo de ponderação foi a multiplicação do inverso da probabilidade de ter participado no seguimento em função das variáveis preditoras da participação pelo inverso da probabilidade de receber benefício do PBF, em função das variáveis preditoras do recebimento do benefício (o escore de propensão).

Para verificar o balanceamento entre os grupos (crianças não beneficiárias e beneficiarias do PBF que pertenciam às famílias com renda *per capita* mensal de até R$ 280,00 e/ou classe D/E) em relação às variáveis de ajuste, foram realizados testes por meio da rotina *tebalance summ*, com obtenção das estimativas: diferenças absolutas padronizadas entre as médias (entre −0,2 e 0,2) e razão de variância (entre 0,9 a 1,1)^24^.

Verificou-se se havia área de suporte comum por meio da distribuição do escore de propensão em beneficiários e não beneficiários do PBF em *boxplot*. Foram fixados níveis de significância de 5% e adotados intervalos de 95% de confiança (IC95%). Empregou-se nas análises o pacote estatístico Stata (versão 14.0).

### Aspectos Éticos

Em São Luís, a pesquisa foi aprovada pelo Comitê de Ética em Pesquisa (CEP) do Hospital Universitário da Universidade Federal do Maranhão, por meio do Parecer nº 223/2009-30. Em Ribeirão Preto, foi aprovada pelo CEP do Hospital das Clínicas de Ribeirão Preto e da Faculdade de Medicina de Ribeirão Preto da Universidade de São Paulo, sob o Protocolo nº 4.116/2008.

## RESULTADOS

O percentual de crianças pertencentes a famílias de baixa renda (até R$ 280,00) que não recebiam o benefício do PBF foi maior em Ribeirão Preto (41,3%) do que em São Luís (29,1%) (Tabela 1). Destas crianças, em São Luís, 3,6% tinham renda familiar *per capita* mensal de até R$ 70,00, 22,6% de R$ 71,00 a R$ 140,00, e 73,8% de R$ 141,00 a R$ 280,00. Em Ribeirão Preto, estes percentuais foram de 2,3% até R$ 70,00, 13,7% de R$ 71,00 a R$ 140,00 e 84% de R$ 141,00 a R$ 280,00 (dados não apresentados em tabela).

**Tabela 1:** Percentuais de incompletude vacinal, recebimento do benefício do Programa Bolsa Família e variáveis de ajuste de crianças de baixa renda, de 13 a 35 meses de idade, nas coortes de nascimento BRISA, Ribeirão Preto (SP) e São Luís (MA), Brasil, 2010–2013.

Variáveis			São Luís				Ribeirão Preto			
			N (3.076)^e^	%	N (1.229)^f^	%	N (3.435)^e^	%	N (532)^f^	%
Incompletude vacinal^a^								
	Vacina BCG		17	0,6	9	0,7	72	2,1	10	1,9
	Vacina poliomielite		135	4,4	74	6,0	98	2,8	15	2,8
	Vacina hepatite B		178	5,8	63	5,9	101	2,9	14	2,6
	Vacina tetravalente		251	8,2	103	8,4	155	4,5	17	3,2
	Vacina febre amarela		310	10,1	129	10,5	128	3,7	16	3,0
	Vacina tríplice viral		341	11,1	153	12,5	155	4,5	28	5,3
	Vacina rotavírus humano		591	19,2	287	23,3	227	6,6	54	10,1
	Esquema vacinal infantil^b^		1.045	33,9	460	37,4	422	12,3	81	15,2
Variável de exposição										
	Beneficiário do Programa Bolsa Família									
		Não	1.432	46,5	358	29,1	2.683	78.2	219	41,3
		Sim	1.644	53,5	871	70,9	749	21,8	311	58,7
Variáveis de ajuste										
	Classe econômica^c^									
		A/B	565	18,4	42	3,4	1.597	46,5	69	12,9
		C	1.782	57,9	713	58,0	1.594	46,4	353	66,4
		D/E	729	23,7	474	38,6	244	7.1	110	20,7
	Escolaridade da mãe em anos									
		> 12	419	13,8	27	2,3	745	22,0	9	1,7
		9 a 11	2.244	73,8	951	78,1	2.172	64,1	334	63,9
		0 a 8	380	12,4	239	19,6	470	13,9	179	34,3
	Cor de pele da mãe^d^									
		Branca	539	17,7	151	12,4	2.005	59,2	219	41,9
		Parda	2.089	68,8	888	72,3	1.045	30,9	218	41,7
		Preta	409	13,5	182	14,9	336	9,9	86	16,4

Variáveis			São Luís (n = 1.229)^f^				Ribeirão Preto (n = 532)^f^			
			n (358)^g^	%	n (871)^h^	%	n (219)^g^	%	n (311)^h^	%
Incompletude vacinal^a^										
	Vacina BCG		02	0,6	7	0,8	06	2,7	04	1,3
	Vacina poliomielite		27	7,5	47	5,4	09	4,1	06	1,9
	Vacina hepatite B		21	5,9	42	4,8	08	3,6	06	1,9
	Vacina tetravalente		34	9,5	69	7,9	08	3,6	09	2,9
	Vacina febre amarela		42	11,7	87	9,9	08	3,6	08	2,6
	Vacina tríplice viral		46	12,8	107	12,3	09	4,1	19	6,1
	Vacina rotavírus humano		88	24,6	199	22,8	18	8,2	36	11,6
	Esquema vacinal infantil^b^		134	37,4	326	37,4	27	12,3	54	17,4
Variáveis de ajuste										
	Classe econômica^c^									
		A/B	20	5,6	22	2,6	41	18,7	28	9,0
		C	208	58,1	505	57,9	139	63,5	213	68,5
		D/E	130	36,3	344	39,5	39	17,8	70	22,5
	Escolaridade da mãe em anos									
		> 12	13	3,7	14	1,6	05	2,3	04	1,3
		9 a 11	277	78,2	674	78,1	158	73,5	175	57,4
		0 a 8	64	18,1	175	20,3	52	24,2	126	41,3
	Cor de pele da mãe^d^									
		Branca	50	14,0	101	11,7	110	50,9	108	35,3
		Parda	258	72,3	630	72,9	73	33,8	145	47,4
		Preta	49	13,7	133	15,4	33	15,3	53	17,3

Diferenças entre os somatórios dos valores absolutos e amostra, devido a informações perdidas; BRISA: Brazilian Ribeirão Preto and São Luís Birth Cohort Studies.

a Vacinação incompleta segundo parâmetros do Ministério da Saúde (MS).

b Esquema vacinal infantil incompleto: não ter recebido pelo menos uma dose da vacina BCG, três para hepatite B, três para poliomielite, três da tetravalente, uma para febre amarela, uma da tríplice viral e duas para o rotavírus humano. Vacinas do primeiro ano de vida, que faziam parte do Calendário Nacional de Vacinação da Criança no início de 2010.

c Classe econômica segundo Associação Brasileira de Estudos e Pesquisas (Abep).

d Cor de pele da mãe (autorreferida).

e Total de crianças do momento do seguimento em menores de 3 anos de vida, com caderneta de saúde visualizada.

f Crianças pertencentes a famílias com renda *per capita* mensal de até R$ 280,00.

g Crianças pertencentes a famílias com renda *per capita* mensal de até R$ 280,00 não beneficiárias do PBF.

h Crianças pertencentes a famílias com renda *per capita* mensal de até R$ 280,00 beneficiárias do PBF.

Em São Luís, o percentual de incompletude do esquema vacinal infantil nas crianças de baixa renda foi igual entre beneficiários (37,4%) e não beneficiários (37,4%), enquanto em Ribeirão Preto foi maior entre beneficiários (17,4%) em relação aos não beneficiários (12,3%) (Tabela 1).

Tanto em São Luís como em Ribeirão Preto, entre as crianças pertencentes a famílias de baixa renda (renda familiar *per capita* de até R$ 280,00), ser beneficiário do PBF não teve efeito no esquema vacinal infantil, segundo ponderação pelo inverso da probabilidade de exposição (São Luís - coeficiente: −0,01; IC95% −0,07 – 0,04; p = 0,708; e Ribeirão Preto - coeficiente: 0,04; IC95% −0,02 – 0,10; p = 0,218) e pareamento por escore de propensão (São Luís - coeficiente: −0,01; IC95% −0,07 – 0,05; p = 0,744; e Ribeirão Preto - coeficiente: 0,04; IC95% −0,02 – 0,10; p = 0,231).

Entre as crianças pertencentes às famílias das classes D/E, nos dois municípios, ser beneficiário do PBF também não teve efeito no esquema vacinal infantil, segundo ponderação pelo inverso da probabilidade de exposição (São Luís - coeficiente: −0,04; IC95% −0,11 – 0,03; p = 0,288; e Ribeirão Preto - coeficiente: −0,01; IC95% −0,11 – 0,08; p = 0,827) e pareamento por escore de propensão (São Luís - coeficiente: −0,04; IC95% −0,11 – 0,03; p = 0,312; e Ribeirão Preto - coeficiente: −0,01; IC95% −0,11 – 0,09; p = 0,820).

Também não houve efeito do PBF na vacinação infantil quando se analisou cada vacina isoladamente (vacina BCG, hepatite B, rotavírus humano, poliomielite, tetravalente, tríplice viral e febre amarela) (Tabela 2).

**Tabela 2:** Estimativas para o efeito de ser beneficiário do Programa Bolsa Família na vacinação de crianças de baixa renda (renda familiar *per capita* mensal de até R$ 280,00/classe econômica D/E), de 13 a 35 meses de idade. Coortes de nascimento BRISA, Ribeirão Preto (SP) e São Luís (MA), Brasil, 2010–2013.

Incompletude vacinal	Crianças pertencentes a famílias com renda *per capita* mensal de até R$ 280,00 Beneficiárias do Programa Bolsa Família							
	São Luís (N = 1.229)				Ribeirão Preto (N = 532)			
	Ponderação pelo inverso da probabilidade de exposição		Pareamento por escore de propensão		Ponderação pelo inverso da probabilidade de exposição		Pareamento por escore de propensão	
	Coeficiente (IC95%)	p	Coeficiente (IC95%)	p	Coeficiente (IC95%)	p	Coeficiente (IC95%)	p
Esquema vacinal infantil^a^	-0,01 (-0,07 – 0,04)	0,708	-0,01 (-0,07 – 0,05)	0,744	0,04 (-0,02 – 0,10)	0,218	0,04 (-0,02 – 0,10)	0,231
Vacina BCG^b^	-0,01 (-0,01 – 0,00)	0,634	-0,01 (-0,01 – 0,00)	0,795	0,01 (-0,01 – 0,04)	0,265	0,01 (-0,01 – 0,04)	0,264
Vacina hepatite B^c^	0,01 (-0,01 – 0,04)	0,405	0,01 (-0,00 – 0,04)	0,370	0,01 (-0,01 – 0,04)	0,291	0,01 (-0,01 – 0,04)	0,368
Vacina rotavírus^d^	0,02 (-0,02 – 0,08)	0,289	0,03 (-0,02 – 0,08)	0,260	-0,02 (-0,07 – 0,02)	0,365	-0,02 (-0,07 – 0,02)	0,357
Vacina poliomielite^e^	0,02 (-0,00 – 0,05)	0,101	0,02 (-0,00 – 0,05)	0,097	0,01 (-0,01 – 0,04)	0,303	0,01 (-0,01 – 0,04)	0,305
Vacina tetravalente^f^	0,02 (-0,01 – 0,05)	0,260	0,02 (-0,01 – 0,06)	0,196	-0,01 (-0,03 – 0,03)	0,963	-0,01 (-0,03 – 0,03)	0,975
Vacina tríplice viral^g^	0,01 (-0,03 – 0,05)	0,595	0,01 (-0,03 – 0,05)	0,594	-0,01 (-0,05 – 0,02)	0,425	-0,01 (-0,05 – 0,03)	0,566
Vacina febre amarela^h^	0,02 (-0,01 – 0,06)	0,210	0,02 (-0,01 – 0,06)	0,181	0,01 (-0,02 – 0,04)	0,516	0,01 (-0,02 – 0,04)	0,517

Incompletude vacinal	Crianças pertencentes às famílias das classes D/E Beneficiárias do Programa Bolsa Família							
	São Luís (N = 729)				Ribeirão Preto (N = 244)			
	Ponderação pelo inverso da probabilidade de exposição		Pareamento por escore de propensão		Ponderação pelo inverso da probabilidade de exposição		Pareamento por escore de propensão	
	Coeficiente (IC95%)	p	Coeficiente (IC95%)	p	Coeficiente (IC95%)	p	Coeficiente (IC95%)	p
Esquema vacinal infantil^a^	-0,04 (-0,11 – 0,03)	0,288	-0,04 (-0,11 – 0,03)	0,312	-0,01 (-0,11 – 0,08)	0,827	-0,01 (-0,11 – 0,09)	0,820
Vacina BCG^b^	0,01 (-0,00 – 0,01)	0,556	0,01 (-0,00 – 0,01)	0,528	0,02 (-0,00 – 0,05)	0,080	0,02 (-0,00 – 0,05)	0,082
Vacina hepatite B^c^	0,01 (-0,02 – 0,04)	0,551	0,01 (-0,02 – 0,04)	0,571	0,03 (-0,01 0,07)	0,197	0,02 (-0,01 – 0,07)	0,231
Vacina rotavírus^d^	0,03 (-0,03 – 0,11)	0,263	0,03 (-0,03 – 0,11)	0,258	0,02 (-0,06 – 0,10)	0,611	0,02 (-0,06 – 0,11)	0,572
Vacina poliomielite^e^	0,01 (-0,02 – 0,05)	0,568	0,01 (-0,02 – 0,05)	0,606	0,03 (-0,00 – 0,07)	0,053	0,03 (-0,00 – 0,07)	0,055
Vacina tetravalente^f^	0,03 (-0,01 – 0,08)	0,160	0,03 (-0,01 – 0,07)	0,187	0,01 (-0,04 – 0,07)	0,555	0,01 (-0,04 – 0,07)	0,631
Vacina tríplice viral^g^	0,03 (-0,01 – 0,08)	0,213	0,03 (-0,01 – 0,09)	0,187	0,06 (-0,00 – 0,11)	0,062	0,06 (-0,00 – −0,11)	0,064
Vacina febre amarela^h^	0,05 (-0,00 – 0,11)	0,061	0,05 (-0,00 – 0,11)	0,058	0,02 (-0,01 0,05)	0,225	0,02 (-0,01 – 0,05)	0,238

BRISA: *Brazilian Ribeirão Preto and São Luís Birth Cohort Studies*; IC95% intervalo de confiança com nível de significância de 5%.

a Esquema vacinal infantil incompleto: não ter recebido pelo menos uma dose da vacina BCG, três para hepatite B, três para poliomielite, três da tetravalente, uma para febre amarela, uma da tríplice viral e duas para o rotavírus humano. Vacinas do primeiro ano de vida, que faziam parte do Calendário Nacional de Vacinação da Criança no início de 2010.

b Vacina BCG incompleta: não ter recebido pelo menos uma dose.

c Vacina hepatite B incompleta: não ter recebido pelo menos três doses.

d Vacina rotavírus humano incompleta: não ter recebido pelo menos duas doses.

e Vacina poliomielite incompleta: não ter recebido pelo menos três doses.

f Vacina tetravalente incompleta: não ter recebido pelo menos três doses.

g Vacina tríplice viral incompleta: não ter recebido pelo menos uma dose.

h Vacina febre amarela incompleta: não ter recebido pelo menos uma dose.

Nos dois municípios, ao se analisar as crianças cujas famílias tinham renda familiar *per capita* mensal de até R$ 140,00 como critério de elegibilidade ao benefício do PBF (segundo o Ministério do Desenvolvimento Social), ou o total de crianças da amostra, ser beneficiário do PBF também não teve efeito no esquema vacinal infantil e para cada vacina isoladamente.

Para o esquema vacinal infantil, alcançou-se o balanceamento entre os grupos de beneficiários e não beneficiários do PBF pelos dois critérios de elegibilidade utilizados para todas as variáveis de ajuste, sugerindo haver permutabilidade entre os grupos em relação às variáveis observadas (Tabela 3). Também obteve-se balanceamento entre os grupos nas demais análises. O *boxplot* demonstrou haver área de suporte comum entre beneficiários e não beneficiários do PBF.

**Tabela 3:** Diferenças padronizadas e razões de variância das variáveis de ajuste para estimar o efeito de ser beneficiário do Programa Bolsa Família na vacinação de crianças pertencentes a famílias com renda *per capita* mensal de até R$ 280,00/classe econômica D/E, de 13 a 35 meses de idade, nas coortes de nascimento BRISA, São Luís (MA) e Ribeirão Preto (SP), Brasil, 2010-2013.

Variáveis de ajuste		Crianças pertencentes a famílias com renda *per capita* mensal de até R$ 280,00 beneficiárias e não beneficiárias do Programa Bolsa Família					
		Bruto		Ponderação pelo inverso da probabilidade de exposição		Pareamento por escore de propensão	
		Diferença padronizada	Razão de variância	Diferença padronizada	Razão de variância	Diferença padronizada	Razão de variância
São Luís (n = 1.229)							
Classe econômica^a^							
	A/B						
	C	-0,01	1,00	-0,00	1,00	-0,00	1,00
	D/E	0,07	1,03	0,00	1,00	0,00	1,00
Escolaridade da mãe em anos							
	> 12						
	9 a 12	-0,00	1,00	-0,00	1,00	-0,00	1,00
	0 a 8	0,05	1,09	0,00	1,00	0,00	1,00
Cor de pele da mãe^b^							
	Branca						
	Parda	0,01	0,98	0,00	0,99	0,00	0,99
	Preta	0,04	1,08	-0,00	0,99	0,00	1,00
Ribeirão Preto (n = 532)							
Classe econômica^a^							
	A/B						
	C	0,11	0,92	-0,00	1,00	-0,01	1,00
	D/E	0,09	1,15	0,00	1,00	0,00	1,00
Escolaridade da mãe em anos							
	> 12						
	9 a 12	-0,34	1,24	0,01	0,99	-0,00	1,00
	0 a 8	0,36	1,31	-0,01	0,99	0,01	1,00
Cor de pele da mãe^b^							
	Branca						
	Parda	0,28	1,11	-0,01	0,99	-0,00	0,99
	Preta	0,05	1,10	0,01	1,02	0,00	1,00

Variáveis de ajuste		Crianças pertencentes a famílias das classes D e beneficiárias e não beneficiárias do Programa Bolsa Família					
		Bruto		Ponderação pelo inverso da probabilidade de exposição		Pareamento por escore de propensão	
		Diferença padronizada	Razão de variância	Diferença padronizada	Razão de variância	Diferença padronizada	Razão de variância
São Luís (n = 729)							
Escolaridade da mãe em anos							
	> 12						
	9 a 12	0,01	0,98	0,00	0,99	0,00	1,00
	0 a 8	0,05	1,06	-0,00	0,99	0,00	1,00
Cor de pele da mãe^2^							
	Branca						
	Parda	-0,02	1,02	-0,00	0,99	-0,00	1,00
	Preta	0,08	1,18	0,00	0,99	0,00	1,00
Ribeirão Preto (n = 244)							
Escolaridade da mãe em anos							
	> 12						
	9 a 12	-0,28	1,23	-0,00	1,00	-0,00	1,00
	0 a 8	0,30	1,28	0,00	1,00	0,00	1,00
Cor de pele da mãe^2^							
	Branca						
	Parda	0,24	0,99	0,00	1,00	0,02	1,00
	Preta	0,14	1,32	0,00	1,00	0,02	1,00

BRISA: *Brazilian Ribeirão Preto and São Luís Birth Cohort Studies.*

a Classe econômica segundo Associação Brasileira de Estudos e Pesquisas (Abep).

b Cor de pele da mãe (autorreferida).

## DISCUSSÃO

Neste estudo, observou-se que ser beneficiário do PBF não teve efeito sobre a vacinação infantil em crianças pertencentes a famílias de baixa renda, tanto em São Luís quanto em Ribeirão Preto.

Uma das limitações da pesquisa é o viés de seleção, por causa de perdas ocorridas no seguimento da coorte. Entretanto, buscou-se reduzir esse possível viés por meio da ponderação das estimativas também pelo inverso da probabilidade de participação no seguimento, além do escore de propensão. Outra limitação possível é o viés de confundimento por variável omitida. Apesar do uso do gráfico acíclico direcionado para representar o modelo teórico, se esse modelo não refletir a realidade, o ajuste realizado utilizando as variáveis identificadas pelo critério da porta de trás pode não ter sido suficiente para remover o confundimento. Contudo, consideramos pequena a possibilidade de confundimento por variável omitida ou inadequadamente especificada (por exemplo, ocupação do chefe de família), por termos incluído no ajuste três variáveis de mensuração do nível socioeconômico, que é o principal confundidor da associação estudada.

Dentre os pontos fortes do estudo, destaca-se a análise comparativa entre dois municípios com condições socioeconômicas diferentes, o que dá mais consistência aos resultados.

Empregou-se pareamento com base no escore de propensão e ponderação pelo inverso da probabilidade de exposição para avaliar o efeito do PBF na vacinação de crianças de baixa renda^3^^,^^4^ e reduzir viés de confundimento. Diferentemente da ponderação pelo inverso da probabilidade de exposição, o pareamento por escore de propensão tende a apresentar maior validade interna e menor validade externa dos dados^18^.

Apesar do menor percentual de crianças de baixa renda não contempladas pelo PBF em São Luís (29,1%) em comparação com Ribeirão Preto (41,3%), o percentual de incompletude do esquema vacinal infantil, também em crianças de baixa renda, foi maior em São Luís (37,4%) em relação a Ribeirão Preto (15,2%). O percentual de incompletude, entretanto, foi elevado nos dois municípios. Geralmente, regiões comparativamente mais pobres, como São Luís, têm mais famílias de baixa renda contempladas pelo PBF^25^ e maior incompletude vacinal^8^^–^^11^^,^^13^^,^^14^^,^^16^^,^^26^.

A incompletude vacinal é maior nas crianças de baixa renda, e receber o benefício do PBF não teve influência sobre a vacinação infantil, seja para cada vacina isoladamente ou para o conjunto delas, nos dois municípios. O cumprimento do Calendário Nacional de Vacinação da Criança é uma das condicionalidades para manter o recebimento do benefício do PBF pelas crianças^2^. Contudo, esse monitoramento pode não estar sendo efetivo^25^. O PBF parece não estar conseguindo melhorar esse indicador de saúde tão importante, que é a vacinação infantil^4^. Ou a condicionalidade não está sendo observada adequadamente, ou talvez somente ela, isoladamente, não seja suficiente para garantir a cobertura vacinal se outras ações não forem implementadas, tais como a ampliação da atenção primária e a disponibilidade de vacinas nas unidades básicas de saúde.

Outro estudo, também com crianças de baixa renda e com representatividade para três grandes áreas do Brasil (regiões Nordeste, Sudeste/Sul e Norte/Centro-Oeste), não encontrou influência do PBF na vacinação infantil^4^. No estudo, foi utilizado escore de propensão na análise estatística. Entretanto, a pesquisa foi realizada logo no segundo ano de implantação do Bolsa Família (2005), quando o programa ainda não havia passado por momentos de grande expansão, e o monitoramento das condicionalidades de saúde ainda estava sendo implementado. O presente estudo foi realizado de 2011 a 2013, quando o programa já estava consolidado.

Outros estudos encontraram resultados diferentes dos da presente investigação, com associação positiva entre receber benefício de programa de transferência condicionada de renda e maior cobertura vacinal infantil^3^^,^^27^. O estudo de Shei et al.^3^ também avaliou o PBF e fez uso de escore de propensão em suas análises estatísticas, mas, diferentemente deste estudo, não tinha abrangência municipal, pois ficou restrito a uma comunidade de baixa renda de Salvador. Os autores ainda ressaltaram que participantes da pesquisa estavam ligados a uma unidade de saúde local, o que pode ter favorecido o acesso aos serviços de saúde e, com isso, um melhor acompanhamento das condicionalidades, incluindo a vacinação infantil.

Pesquisa demográfica e de saúde realizada na Índia, de 2007 a 2008, com crianças de 12 a 23 meses, fez uso de escore de propensão e identificou aumento nas taxas de vacinação infantil em crianças beneficiárias de programa de transferência condicionada de renda. No estudo indiano, foram consideradas também informações relatadas de vacinação, além de dados das crianças que apresentavam comprovação da situação vacinal. O efeito do programa de transferência condicionada de renda na vacinação tendeu a desaparecer quando foram considerados apenas os dados de crianças com cartões de imunização^27^, sugerindo que a associação positiva observada tenha ocorrido devido a viés de aferição. No presente estudo, foram considerados apenas os dados de vacinação anotados no cartão da criança.

Um programa de transferência condicionada de renda que tem demostrado possível aumento na utilização de serviços de saúde preventivos, incluindo a vacinação infantil^28^, é o programa Oportunidades, no México, que melhorou resultados de saúde, crescimento e desenvolvimento infantil. O desempenho do programa resulta de um controle mais efetivo das condicionalidades, incluindo aquelas relacionadas à saúde, por meio de um estruturado sistema de informação para acompanhar as famílias beneficiárias. As transferências de renda são bimestrais, mas só ocorrem se as condicionalidades forem cumpridas pelos beneficiários^29^.

No presente estudo, como demonstramos, ser beneficiário do PBF não influenciou nos percentuais de vacinação de crianças de baixa renda em dois municípios brasileiros situados em duas regiões com diferentes condições socioeconômicas. Portanto, é importante melhorar tanto o acompanhamento da condicionalidade do programa quanto o monitoramento da situação vacinal, uma vez que os percentuais de incompletude vacinal em crianças beneficiárias do PBF foram elevados.
